# End-tidal to arterial carbon dioxide gradient is associated with increased mortality in patients with traumatic brain injury: a retrospective observational study

**DOI:** 10.1038/s41598-021-89913-x

**Published:** 2021-05-17

**Authors:** Pascal Doppmann, Lorenz Meuli, Stephen J. M. Sollid, Miodrag Filipovic, Jürgen Knapp, Aristomenis Exadaktylos, Roland Albrecht, Urs Pietsch

**Affiliations:** 1 Department of Anaesthesiology and Intensive Care Medicine, Cantonal Hospital St, Rorschacher Strasse 95, 9007 GallenSt. Gallen, Switzerland; 2 Department of Vascular Surgery, University Hospital Zurich, Zurich, Switzerland; 3 Norwegian Air Ambulance Foundation, Oslo, Norway; 4 Department of Anaesthesiology and Pain Medicine, Inselspital, Bern University Hospital, University of Bern, Bern, Switzerland; 5 Department of Emergency Medicine, Inselspital, Bern University Hospital, University of Bern, 3010 FreiburgstrasseBern, Switzerland; 6 Swiss Air-Ambulance, Rega (RettungsFlugwacht/Guarde Aérienne), Postfach 1414, 8058 Zurich, Switzerland

**Keywords:** Diseases, Medical research

## Abstract

Early definitive airway protection and normoventilation are key principles in the treatment of severe traumatic brain injury. These are currently guided by end tidal CO_2_ as a proxy for PaCO_2_. We assessed whether the difference between end tidal CO_2_ and PaCO_2_ at hospital admission is associated with in-hospital mortality. We conducted a retrospective observational cohort study of consecutive patients with traumatic brain injury who were intubated and transported by Helicopter Emergency Medical Services to a Level 1 trauma center between January 2014 and December 2019. We assessed the association between the CO_2_ gap—defined as the difference between end tidal CO_2_ and PaCO_2_—and in-hospital mortality using multivariate logistic regression models. 105 patients were included in this study. The mean ± SD CO_2_ gap at admission was 1.64 ± 1.09 kPa and significantly greater in non-survivors than survivors (2.26 ± 1.30 kPa vs. 1.42 ± 0.92 kPa, p < .001). The correlation between EtCO_2_ and PaCO_2_ at admission was low (Pearson's r = .287). The mean CO_2_ gap after 24 h was only 0.64 ± 0.82 kPa, and no longer significantly different between non-survivors and survivors. The multivariate logistic regression model showed that the CO_2_ gap was independently associated with increased mortality in this cohort and associated with a 2.7-fold increased mortality for every 1 kPa increase in the CO_2_ gap (OR 2.692, 95% CI 1.293 to 5.646, p = .009). This study demonstrates that the difference between EtCO_2_ and PaCO_2_ is significantly associated with in-hospital mortality in patients with traumatic brain injury. EtCO_2_ was significantly lower than PaCO_2_, making it an unreliable proxy for PaCO_2_ when aiming for normocapnic ventilation. The CO2 gap can lead to iatrogenic hypoventilation when normocapnic ventilation is aimed and might thereby increase in-hospital mortality.

## Introduction

Treatment recommendations in traumatic brain injury (TBI) include early definitive airway protection as well as normoventilation with a target arterial partial pressure of CO_2_ (PaCO_2_) of 4.6–5.9 kPa (35–45 mmHg)^1,2^. The effects of hypo- or hyperventilation on cerebral blood flow (CBF), with the potential for hypoxemia or hyperemia of cerebral tissue and their negative impact on outcome, have been widely studied^3–7^. Using PaCO_2_ to monitor ventilation requires arterial blood gas (ABG) analyses, but the necessary lab equipment is not yet widely available in the prehospital environment. Therefore end-tidal CO_2_ (EtCO_2_) determined by capnography has been used as a surrogate marker to estimate PaCO_2_ assuming a reliable correlation between EtCO_2_ and PaCO_2_^8^.

Capnography is considered the gold standard, both to determine correct placement of a definitive airway and to guide ventilation during emergency care^9,10^. The assumed correlation between EtCO_2_ and PaCO_2_ has been known to be accompanied by a tension difference of CO_2_ ranging anywhere between 0.26 and 0.66 kPa (2 and 5 mmHg) in otherwise healthy individuals undergoing anesthesia^11–16^. However, major trauma accompanying TBI can negatively influence ventilation and perfusion, making the interpolation of PaCO_2_ from EtCO_2_ in trauma patients unreliable^17–19^. As expected, subgroup analyses have shown the best correlation between EtCO_2_ and PaCO_2_ in isolated TBI when compared to other trauma patients^20^.

The primary aim of this study is to describe the correlation between EtCO_2_ and PaCO_2_ at the time of admission in patients hospitalized with TBI. Furthermore, we investigated the predictive value of tension difference of CO_2_ between EtCO_2_ and PaCO_2_ (CO_2_ gap) for in-hospital mortality.

## Methods

### Study participants, setting and ethics approval

This retrospective observational single-center cohort study included all consecutive patients with TBI who were intubated on the scene and transported by the helicopter emergency medical service (HEMS) (Swiss Air-Rescue, Rega) to a Level 1 trauma center (Kantonsspital St. Gallen, Switzerland) between January 1st of 2014 and December 31st of 2019. Exclusion criteria were patients who were not intubated before admission, patients with traumatic injuries requiring intubation for other reasons than TBI, and secondary transport missions including patients with traumatic brain injury who were transported from another hospital to this trauma center.

The local ethics committee of St. Gallen (EKOS) granted permission to use patient data without individual consent according to the federal act on research involving human beings and the ordinance on human research with the exception of clinical trials. The permission also covered the use of patient data regarding the HEMS operation (EKOS St. Gallen 7.7.2020, BASEC Nr. 2020-01737 EKOS 20/122).

### Data and definitions

Baseline characteristics of patients were obtained from electronic hospital records. Laboratory findings were obtained by automated retrieval using the unique patient identification number in the hospital records. EtCO_2_ was measured using main-stream capnographs (ZOLL Medical Corporation, Chelmsford, USA). Information on the ventilator settings at admission was prospectively entered into the patients' electronic hospital records.

Outcome information (i.e., survival status) was documented prospectively as part of the routine electronic hospital records and obtained from the corresponding record.

The Injury Severity Score Thorax was determined at admission. EtCO_2_, systolic blood pressure, pulse and SpO_2_ were recorded on admission to the Emergency Room (ER) as well as 24 h after admission.

### Statistics

Patients’ characteristics were summarized and presented in tables. Continuous variables were summarized by mean ± SD (standard deviation) if normally distributed or by median and IQR (interquartile range) if skewed. Normality was tested using the Shapiro–Wilk test. Categorical variables were summarized with counts and percentages for each level of the variable. Outliers were assessed using the Grubbs test for continuous variables if normally distributed.

Correlation between EtCO_2_ and PaCO_2_ was assessed using Pearson’s correlation coefficient and visualized using a scatter plot. Disagreement between EtCO_2_ and PaCO_2_ was visualized using a Bland–Altman plot^21^. To assess the impact of the time interval between the initial arterial blood gas sample and the first recorded EtCO_2_ at admission, a linear regression was conducted.

Differences in the CO_2_ gap between survivors and non-survivors were tested using the Mann–Whitney-Wilcoxon Test. The association between the CO_2_ gap and the in-hospital mortality was further assessed using univariate and multivariable logistic regression models. To minimize confounding, variables potentially associated with the respiratory system and in-hospital mortality were defined a priori based on a literature review and clinical experience^22^. The variables included age, heart rate, systolic blood pressure, peripheral capillary oxygen saturation, pressure of oxygen in arterial blood (paO2), and severity of chest injury documented by the ISS (Injury Severity Score) thoracic sub-score. All variables were coded as continuous variables. Complete case analyses were performed due to the low number of missing data and therefore the low risk of bias.

Two-sided p-values of < 0.05 were considered as statistically significant. All statistical analyses were performed using R Studio 3.6.0 on macOS 10.15.7.

### Ethics approval and consent to participate

The local ethics committee of St. Gallen (EKOS) granted permission to use patient data without individual consent according to the federal act on research involving human beings and the ordinance on human research with the exception of clinical trials. The permission also covered the use of patient data regarding the HEMS operation (EKOS St. Gallen 7.7.2020, BASEC Nr. 2020–01737 EKOS 20/122).

### Consent for publication

Consent for publication was waived as per the ethics approval.

## Results

This study adheres to the STROBE Statement (Strengthening the Reporting of Observational Studies in Epidemiology)^23^. From January 2014 to December 2019 a total of 181 patients were admitted to our trauma center by HEMS after TBI and intubation. Seventy-six patients were excluded. Reasons were mechanisms of injury besides TBI, an alternate reason for unconsciousness, missing ISS, EtCO_2_ or PaCO_2_ data, or early extubation in the ER.

Of the 105 patients admitted to the ICU, 28 (27%) died and 77 (73%) were discharged alive. Information on neurological function at discharge was not available.

The patients’ baseline characteristics are displayed in Table 1. Of note, non-survivors were on average more than 20 years older than survivors and had a lower PaO_2_ in the initial blood gas samples, p < 0.001.

**Table 1:** Baseline characteristics of patients.

Variable	Overall n = 105	Survivors n = 77	Non-survivors n = 28
Age, years	49.5 ± 22.6	43.4 ± 21.0	66.3 ± 18.1
Age < 18 years, n (%)	9 (9)	8 (10)	1 (4)
Male gender, n (%)	71 (68)	51 (66)	20 (71)
ISS Total, median (IQR)	25 (17 to 34)	25 (14 to 34)	31 (25 to 38)
ISS = 75, n (%)	1 (1)	0	1 (4)
ISS Thorax, median (IQR)	0 (0 to 2)	0 (0 to 2)	0 (0 to 2)
Missing (ISS Thorax), n (%)	12 (11)	9 (12)	3 (11)
**Cardiopulmonary parameters**			
Systolic BP, mmHg	127 ± 33	127 ± 29	125 ± 43
Diastolic BP, mmHg	78 ± 28	80 ± 30	72 ± 21
Pulse, bpm	93 ± 26	92 ± 24	93 ± 32
SpO_2_, median (IQR)	100 (98 to 100)	100 (98 to 100)	99.5 (94.8 to 100)
Temperature, °C	35.9 ± 0.93	35.9 ± 0.93	35.9 ± 0.98
Missing, n (%)	16	11	5
**Arterial blood gas**			
pH, median (IQR)	7.31 (7.26 to 7.35)	7.31 (7.28 to 7.36)	7.31 (7.19 to 7.33)
BE, mmol/l median (IQR)	− 3.6 (− 6.4 to − 1.9)	− 3.6 (− 5.8 to − 1.7)	− 4.9 (− 8.4 to − 2.3)
HCO_3_^−^, mmol/l median (IQR)	21.9 (19.9 to 23.6)	21.5 (20.5 to 23.6)	21.1 (19.6 to 23.4)
Lactate, mmol/l median (IQR)	2.0 (1.2 to 3.4)	1.9 (1.2 to 2.7)	2.8 (1.4 to 4.5)
PaCO_2_, kPa median (IQR) *	6.0 (5.5 to 6.8)	5.9 (5.5 to 6.6)	6.4 (5.7 to 6.8)
PaO_2_, kPa median (IQR)	28.2 (17.6 to 48.9)	34.3 (19.6 to 51.5)	23.1 (14.9 to 29.8)
Hemoglobin, g/l	122 ± 21	122 ± 20	119 ± 24
Glucose, mmol/l median (IQR)	8.0 (6.3 to 10.1)	7.5 (6.0 to 8.9)	10.2 (8.1 to 13.2)

Data was complete if not otherwise specified. Continuous variables are reported as mean ± SD = (standard deviation) if normally distributed and not stated otherwise.

*BP* blood pressure, *IQR* interquartile range, *ISS* injury severity score, *bpm* beats per minute, *SpO*_*2*_ peripheral capillary oxygen saturation.

*PaCO_2_ = same parameter as shown in detail on Table 2 (initial measure).

**Figure 1:**
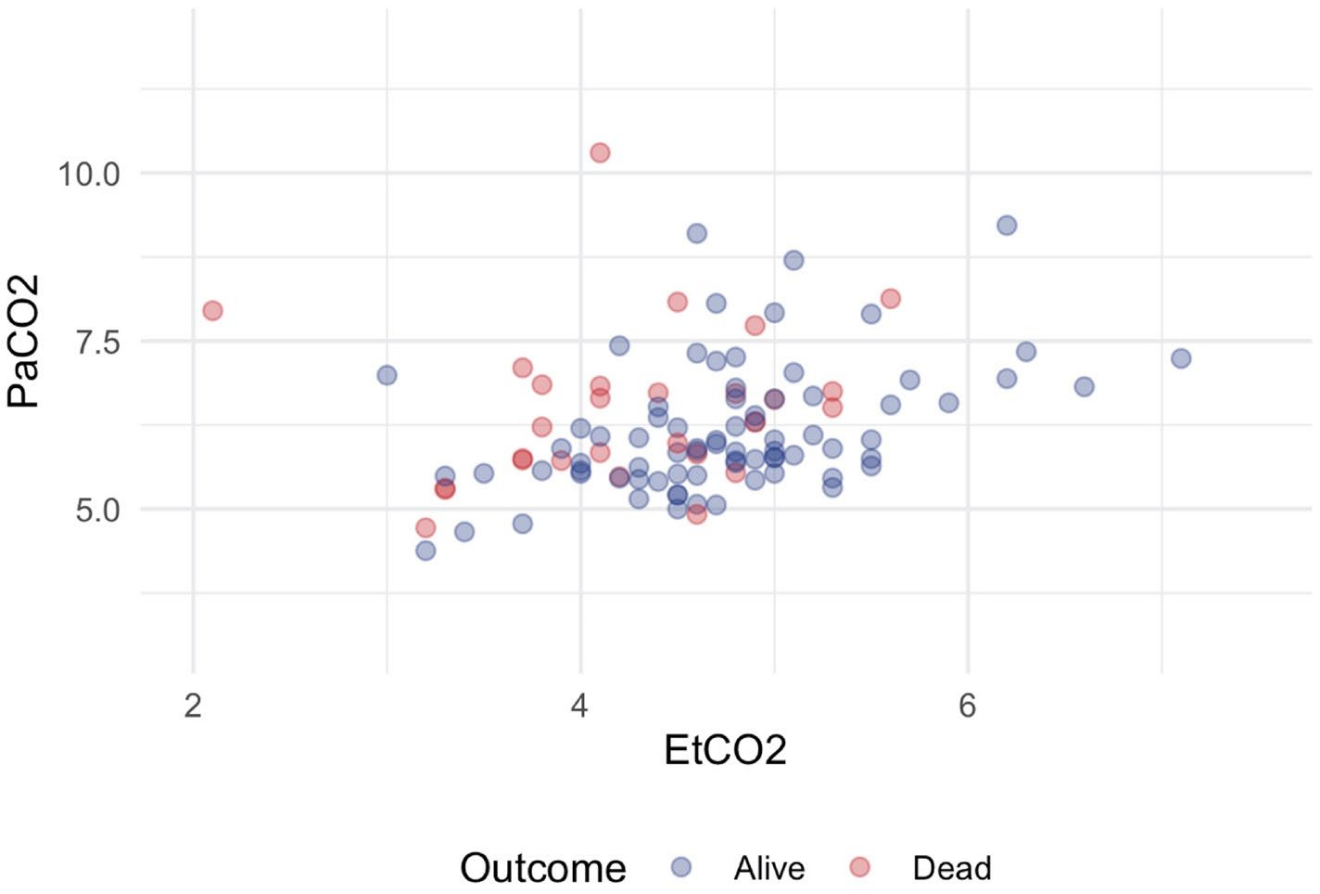
Correlation of PaCO2 and EtCO2. Pearson's correlation coefficient overall r = 0.287, for survivors r = 0.438, for non-survivors r = 0.150. PaCO2 and EtCO2 in kPa. Figure was created using RStudio (2020). RStudio: Integrated Development for R. RStudio, PBC, Boston, MA URL http://www.rstudio.com/ and the package ggplot2 by Wickham H (2016).

**Figure 2:**
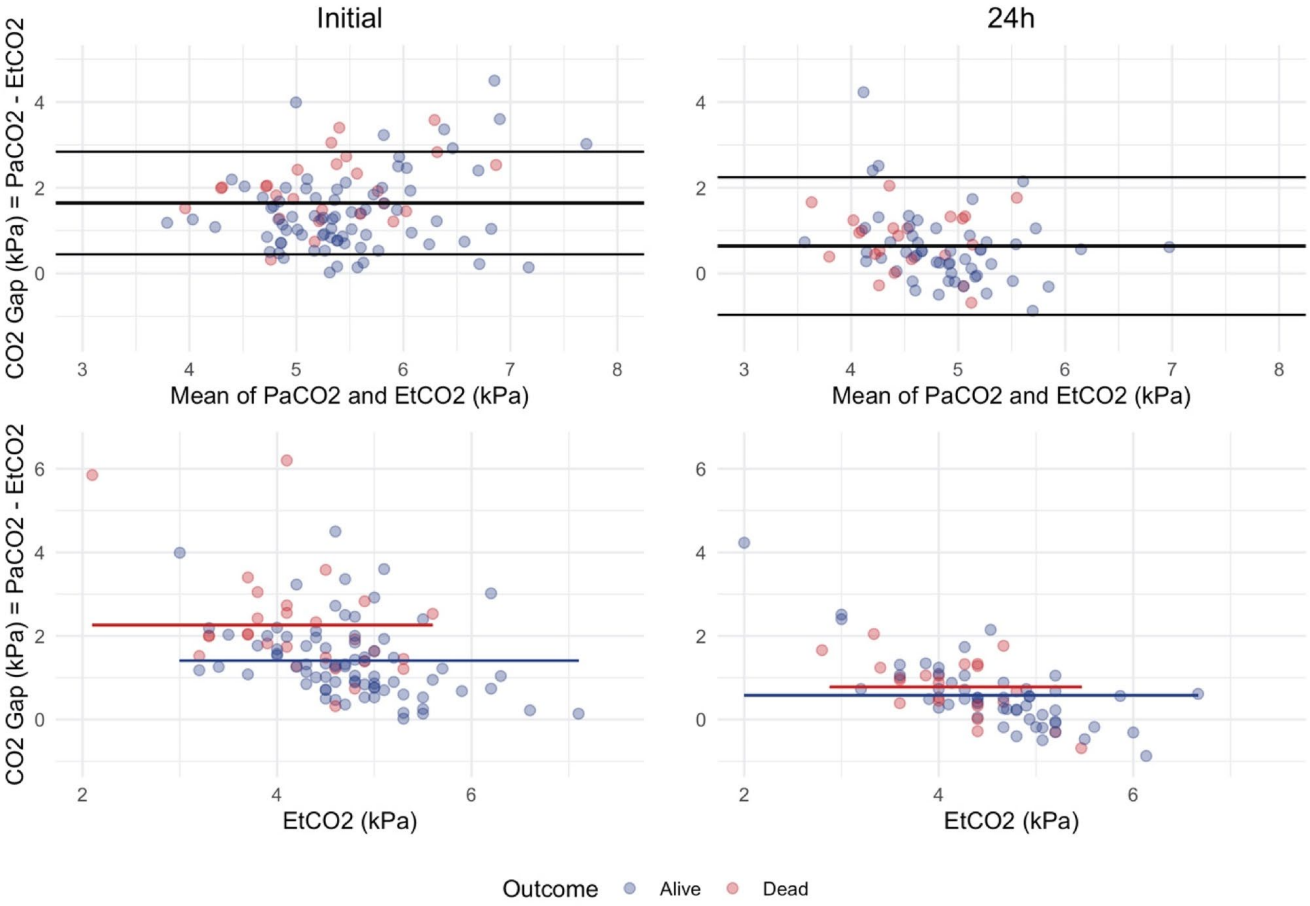
Bland–Altman plots and point plots comparing PaCO2 and EtCO2. Top row: Bland–Altman plots for all available pairs of PaCO2 and EtCO2 at different time points. Bottom row: corresponding point plots for the same data. The red and blue lines illustrate the mean CO2 gap for deceased and surviving patients, respectively. The mean CO2 gap lines are trimmed, illustrating the EtCO2 range for both groups, respectively. Difference between PaCO2 and EtCO2 was highly significant for the initial pairs (p < 0.001) but not for the pairs after 24 h (see Table 2). Figures were created using RStudio (2020). RStudio: Integrated Development for R. RStudio, PBC, Boston, MA URL http://www.rstudio.com/ and the package ggplot2 by Wickham H (2016).

**Figure 3:**
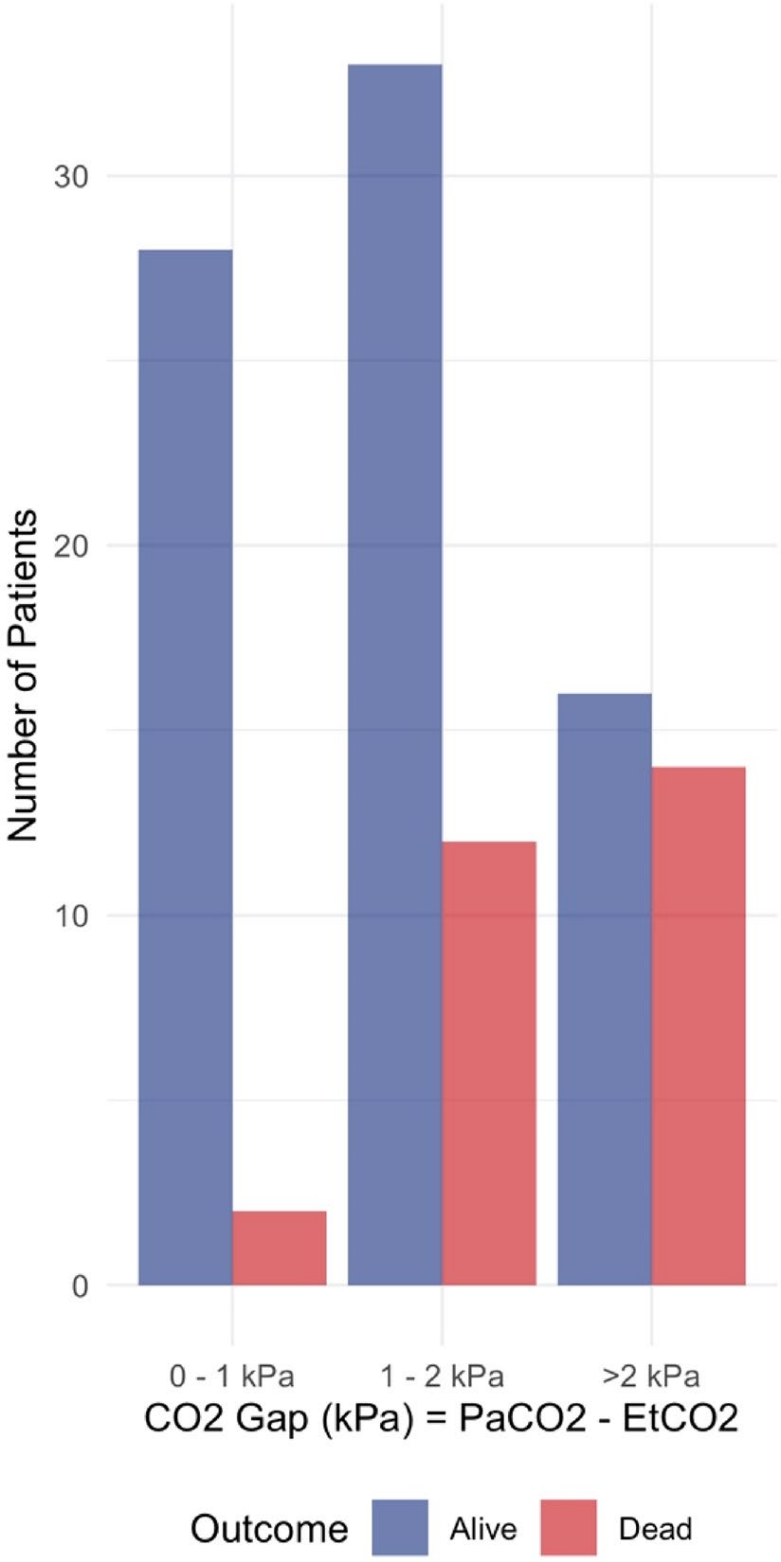
Bar diagram showing survival for CO2 gap groups. Bar diagram showing outcome by groups of CO2 gap measured initially. Figure was created using RStudio (2020). RStudio: integrated development for R. RStudio, PBC, Boston, MA URL http://www.rstudio.com/ and the package ggplot2 by Wickham H (2016).

The correlation between EtCO_2_ and PaCO_2_ at admission was low, Pearson's r = 0.287, Fig. 1. There was a significant difference between EtCO_2_ and PaCO_2_ at admission. The overall mean CO_2_ gap at admission was 1.64 ± 1.09 kPa and significantly larger in non-survivors than survivors, 2.26 ± 1.30 kPa vs. 1.42 ± 0.92 kPa, p < 0.001, see Table 2 and Figs. 2 and 3. The majority of EtCO_2_ and PaCO_2_ pairs were obtained within 30 min, n = 60, 57%. However, there was no significant association between the time intervals of the first arterial blood gas sampling and the first documented EtCO_2_ on the CO_2_ gap in a univariate linear regression, p = 0.165.

**Table 2:** PaCO_2_ and EtCO_2_ pairs at admission and after 24 h.

Variable	Overall n = 105	Survivors n = 77	Non-survivors n = 28	p value
**Initial measures**	105 (100)	76 (99)	28 (100)	
CO_2_ gap, kPa	1.64 ± 1.09	1.42 ± 0.92	2.26 ± 1.30	< 0.001
PaCO_2_, kPa	6.26 ± 1.03	6.17 ± 0.96	6.48 ± 1.18	
EtCO_2_, kPa	4.61 ± 0.78	4.76 ± 0.74	4.23 ± 0.76	
PaCO_2_ < 15 min of EtCO_2_	42 (40)	30 (39)	13 (46)	
PaCO_2_ < 30 min of EtCO_2_	60 (57)	39 (51)	21 (75)	
PaCO_2_ ≥ 30 min of EtCO_2_	45 (43)	38 (49)	7 (25)	
**Measures at 24 h**	75 (71)	53 (70)	22 (79)	
CO_2_ gap, kPa	0.64 ± 0.82	0.58 ± 0.86	0.78 ± 0.70	0.108
PaCO_2_, kPa	5.12 ± 0.60	5.19 ± 0.59	4.91 ± 0.58	
EtCO_2_, kPa	4.46 ± 0.79	4.59 ± 0.82	4.15 ± 0.63	
Hours since admission	18.6 ± 7.8	19 ± 8.0	17.7 ± 7.5	

Data was complete. Numbers are presented with percentages of total in parentheses. Continuous variables are reported as mean ± SD (standard deviation). The CO_2_ gap and the PaCO_2_ variables were skewed; however, the mean ± SD was presented due to the use of these parameters in the Bland–Altman plots. CO_2_ gap = PaO_2_—EtCO_2_.

For the measures at 24 h there was no time delay measurement of EtCO_2_ and PaCO_2_.

The overall CO_2_ gap decreased to 0.64 ± 0.82 kPa at 24 h after admission and was no longer significantly different between non-survivors and survivors, 0.78 ± 0.70 kPa vs. 0.58 ± 0.86, p = 0.108, see Table 2 and Fig. 2.

**Figure 4:**
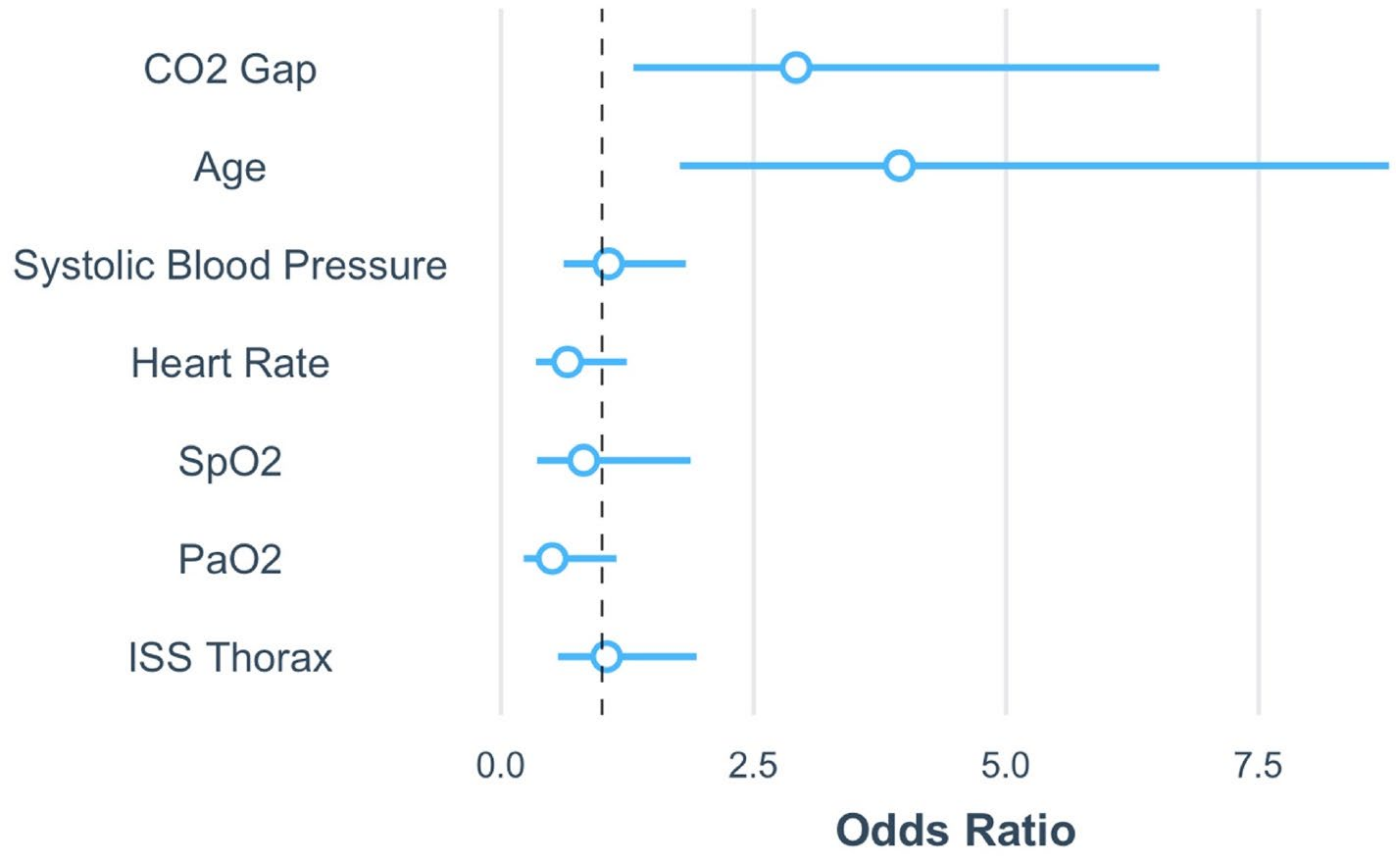
Scaled regression coefficient of the multivariate logistic regression. Illustration of the multivariate logistic regression model summarized on Table 3. Regression coefficients are exponentiated and scaled. The horizontal lines around the dots indicates the 95% confidence interval of the odds ratio. CO2 gap = PaO2—EtCO2. Figure was created using RStudio (2020). RStudio: Integrated Development for R. RStudio, PBC, Boston, MA URL http://www.rstudio.com/. and the package ggplot2 by Wickham H (2016).

The multivariate logistic regression model showed that the CO_2_ gap was independently associated with increased mortality in intubated and mechanically ventilated patients with TBI. For every increase of the CO_2_ gap by 1 kPa, mortality was 2.7 times higher, OR 2.692, 95% CI 1.293–5.646, p = 0.009. Higher age was independently associated with an increased mortality rate as well, OR 1.842 for every increase of 10 years, 95% CI 1.106–2.641, p = 0.001. Systolic blood pressure, heart rate, thoracic trauma, SpO_2_ and PaO_2_ were not associated with survival status in this multivariate model, see Table 3 and Fig. 4. Inclusion of further parameters from the arterial blood gas samples (ABG samples), the total ISS, or other cardiopulmonary parameters in the regression model led to multicollinearity; these parameters were therefore excluded from the final model.

**Table 3:** Logistic regression models of survival.

Variable	Univariate analysis			
	Unadjusted OR	95% CI of OR	Std. Err	p value
CO_2_ gap, kPa	2.044	1.299–3.219	0.232	0.002
Age, year	1.059	1.030–1.088	0.014	< 0.001
Systolic BP, mmHg	0.998	0.985–1.011	0.007	0.750
Pulse, bpm	1.001	0.984–1.018	0.009	0.909
SpO_2_, %	0.948	0.875–1.025	0.039	0.171
PaO_2_, kPa	0.974	0.950–0.998	0.013	0.038
ISS Thorax	1.077	0.796–1.457	0.154	0.629

Variable	Multivariate analysis			
	Adjusted OR	95% CI of OR	Std. err	p value
CO_2_ gap, kPa	2.692	1.283–5.646	0.385	0.009
Age, year	1.063	1.026–1.102	0.018	0.001
Systolic BP, mmHg	1.002	0.986–1.018	0.008	0.822
Pulse, bpm	0.984	0.960–1.009	0.013	0.199
SpO_2_, %	0.960	0.810–1.137	0.106	0.635
PaO_2_, kPa	0.966	0.926–1.007	0.022	0.101
ISS thorax	1.030	0.683–1.554	0.214	0.887

Complete case analysis available for 93 patients (multivariate). Twelve patients were excluded from the multivariate analysis due to missing data (see Table 1). Units of measure and abbreviations as described in Tables 1 and 2.

## Discussion

Our results show that end-tidal capnography is an unreliable tool for monitoring and targeting invasive ventilation at least in the initial treatment of patients with severe TBI. Although the majority of the patients in this study were ventilated within the target range of EtCO_2_ values, many were unwittingly hypercapnic in the first blood gas sample after arriving in the hospital. Our data show a large variability in the calculated CO_2_ gap in this patient cohort and it was more pronounced in patients with lower EtCO_2_. This underestimation of PaCO_2_ when EtCO_2_ was used to guide ventilation caused hypoventilation despite normal EtCO_2_ values. An increased CO_2_ gap and the resulting hypercapnia were associated with increased in-hospital mortality. This underlines the clinical importance of these findings and the need for either a more reliable surrogate parameter for PaCO_2_ estimation or early PaCO_2_ sampling in the prehospital management of patients with TBI.

### The CO_2_ gap

Previous studies have observed that the CO_2_ gap is multifactorial, with possible causes including ventilation-perfusion mismatch, increased dead space, or, shock with impaired perfusion and temperature^11,24^. However, most of these factors influencing the CO_2_ gap are not measurable, detectable or predictable in the initial treatment period in the field or ER. The ability to predict or gauge the CO_2_ gap based on the patient’s condition is consequently limited. In this context the CO_2_ gap might be both, an indicator of severity of injury, and a predictor of impaired survival in patients with severe traumatic brain injury.

Two recent publications investigated the CO_2_ gap in critically ill patients after prehospital emergency anesthesia^25,26^. Their findings are in line with our results and showed only moderate correlation between EtCO_2_ and PaCO_2_, confirming that EtCO_2_ alone should be used with caution to guide ventilation in the critically ill.

This was further strengthened by our data wherein, the CO_2_ gap (visualized as mean bias on the Bland–Altman plots) was more pronounced in patients with lower EtCO_2_ values demonstrating that patients with EtCO_2_ measures within the target range (4.6–5.9 kPa) were unwittingly hypercapnic.

In a cohort of cardiac arrest patients, Suominen et al. showed an association between an increased CO_2_ gap and in-hospital mortality 24 h after return of spontaneous circulation (ROSC). Our data is in line with these findings and reinforces the plausibility of this association by controlling for potential confounding due to shock or hypoperfusion in a multivariate logistic regression model.

### EtCO_2_ as a surrogate marker

PaCO_2_ is considered to be the major determinant of cerebral blood flow (CBF) through its effects on cerebral vascular tone^27^. This reinforces the importance of precise ventilatory control in the initial management of TBI. It is known that even modest hypercapnia can result in substantial increases in ICP and can cause dangerous cerebral ischemia when intracranial compliance is poor^28^. Therefore, we hypothesize that the hypoventilation due to underestimation of the arterial CO_2_ using EtCO_2_ as a surrogate marker leads to impaired CBF and thereby increases mortality.

Recent TBI guidelines rely on the assumption that the CO_2_ gap is approximately 0.5 kPa (3.8 mmHg). However, these assumptions are based on data of individuals undergoing general anesthesia without major comorbidities or trauma^11,29^. In this study, the mean first EtCO_2_ was 4.6 ± 0.78 kPa, whereas the mean PaCO_2_ was 6.26 ± 1.03 kPa and far in excess of the target of 4.5–5.0 kPa. Therefore, relying on EtCO_2_ as a surrogate for PaCO_2_ provides a false sense of security, and providers may not achieve optimal prehospital PaCO_2_. At present, no reliable alternative to direct ABG sampling seems to exist in order to approximate PaCO_2_ reliably.

However, to our best knowledge, there is no data supporting the routine use of point-of-care blood gas analyses in patients mechanically ventilated in the field. This lack of data could be due to the fact that up to now the importance of point-of-care testing in prehospital care has been underestimated, due to the high reliance on proxy markers like EtCO_2_. Further studies on the optimal timing of sampling after intubation and the beginning of mechanical ventilation, as well as the optimal sampling interval, are needed. We postulate that a single ABG sample post-intubation could gauge the individual CO_2_ gap and ensure more reliable EtCO_2_-guided ventilation.

### Factors influencing mortality

Our data showed a significant age difference between survivors and non-survivors. Age was independently and significantly associated with mortality. Besides the fact that age might be a surrogate for unrecognized confounders due to comorbidities that negatively influence mortality, clinical decision-making may also play a role. In daily routine, palliation might be considered at an earlier stage in elderly trauma victims with limited rehabilitation potential, whereas younger trauma patients may receive maximum therapeutic interventions^30^.

In our cohort, systolic blood pressure and ISS thorax scores were not significantly associated with mortality in the multivariate analysis.

### Limitations

This study had several limitations. First, it is a retrospective and single-center cohort study with a limited sample size. However, data was almost complete and multivariate adjustments were performed. Second, in order to increase the number of eligible patients in this study, we included patients who had an ABG sample up to 30 min after hospital arrival. However, a sensitivity analysis showed that the observed gradient between EtCO_2_ and PaCO_2_ was not significantly associated with the time between arterial blood gas sampling and the documented EtCO_2_. Still, it is possible that a proportion of the gradient between EtCO_2_ and PaCO_2_ was due to changes in ventilation settings during this period. Furthermore, if this time difference was longer than 15 min apart there was no second set of hemodynamic data to statistically evaluate at these two different timepoints.

Lastly, ventilation mode selected in the preclinical setting was not taken into account in analysis of the data. We cannot exclude bias through varying influence of ventilation mode on dead space.

## Conclusions

The CO_2_ gap is an inconsistent phenomenon in pre-hospital anesthetized TBI patients, making EtCO_2_ an unreliable proxy for PaCO_2_ when aiming for normocapnic ventilation. The higher-than-expected CO_2_ gap can lead to iatrogenic hypoventilation when normocapnic ventilation is aimed for and might thereby increase in-hospital mortality."

## Data Availability

The datasets used and/or analyzed during the current study are available from the corresponding author on reasonable request.
